# *N*,*N*′-Bis(2,3-dimethoxy­benzyl­idene)propane-1,3-diamine

**DOI:** 10.1107/S1600536809031651

**Published:** 2009-08-29

**Authors:** Karla Fejfarová, Aliakbar Dehno Khalaji, Michal Dušek

**Affiliations:** a Institute of Physics of the ASCR, Na Slovance 2, 182 21 Prague 8, Czech Republic; b Department of Chemistry, Faculty of Science, Golestan University, Gorgan, Iran

## Abstract

The title compound, C_21_H_26_N_2_O_4_, adopts an *E* configuration with respect to the azomethine C=N bonds. The dihedral angle between the two rings is 8.16 (8)°. The crystal structure is stabilized by weak inter­molecular C—H⋯O inter­actions.

## Related literature

For the chemistry of Schiff base derivatives, see: Morshedi *et al.* (2009*a* ▶,*b* ▶); Dehno Khalaji *et al.* (2009 ▶); Khalaji *et al.* (2007 ▶); Wang (2008 ▶); Fun *et al.* (2008 ▶). For their applications, see: Ardizzoia *et al.* (2009 ▶); Gao *et al.* (2003 ▶). For the extinction correction, see: Becker & Coppens (1974 ▶).
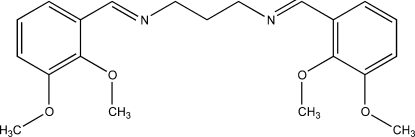


## Experimental

### 

#### Crystal data

C_21_H_26_N_2_O_4_
*M* *_r_* = 370.4
Orthorhombic, 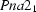

*a* = 15.3079 (3) Å
*b* = 9.2915 (2) Å
*c* = 13.8059 (3) Å
*V* = 1963.66 (7) Å^3^
*Z* = 4
Cu *K*α radiation
μ = 0.71 mm^−1^
*T* = 120 K
0.51 × 0.31 × 0.20 mm

#### Data collection

Oxford Diffraction Xcalibur diffractometer with an Atlas (Gemini ultra Cu) detector
Absorption correction: multi-scan (*CrysAlis Pro*; Oxford Diffraction, 2009 ▶) *T*_min_ = 0.628, *T*_max_ = 0.871
26538 measured reflections
1623 independent reflections
1616 reflections with *I* > 3σ(*I*)
*R*_int_ = 0.021
θ_max_ = 62.4°

#### Refinement

*R*[*F* ^2^ > 2σ(*F* ^2^)] = 0.025
*wR*(*F* ^2^) = 0.077
*S* = 1.97
1623 reflections
244 parameters
H-atom parameters constrained
Δρ_max_ = 0.10 e Å^−3^
Δρ_min_ = −0.09 e Å^−3^

### 

Data collection: *CrysAlis Pro* (Oxford Diffraction, 2009 ▶); cell refinement: *CrysAlis Pro*; data reduction: *CrysAlis Pro*; program(s) used to solve structure: *SIR2002* (Burla *et al.*, 2003 ▶); program(s) used to refine structure: *JANA2006* (Petříček *et al.*, 2007 ▶); molecular graphics: *DIAMOND* (Brandenburg & Putz, 2005 ▶); software used to prepare material for publication: *JANA2006*.

## Supplementary Material

Crystal structure: contains datablocks global, I. DOI: 10.1107/S1600536809031651/bt5030sup1.cif

Structure factors: contains datablocks I. DOI: 10.1107/S1600536809031651/bt5030Isup2.hkl

Additional supplementary materials:  crystallographic information; 3D view; checkCIF report

## Figures and Tables

**Table 1:** Hydrogen-bond geometry (Å, °)

*D*—H⋯*A*	*D*—H	H⋯*A*	*D*⋯*A*	*D*—H⋯*A*
C5—H5⋯O4^i^	0.96	2.59	3.411 (2)	143

Symmetry code: (i) 
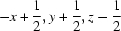
.

**Fig. 1.**
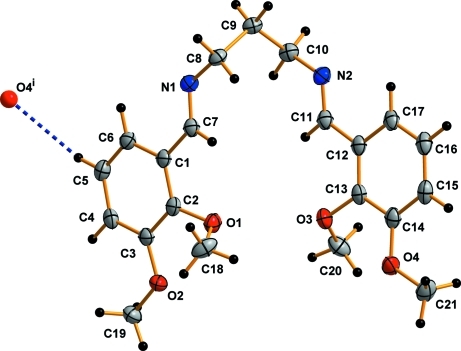
The molecular structure of the title compound with atom-labeling scheme, with hydrogen bond shown as a dashed line. Displacement ellipsoids are drawn at the 50% probability level. [Symmetry codes: (i) 0.5 - x, 1/2 + y, z - 1/2]

**Fig. 2.**
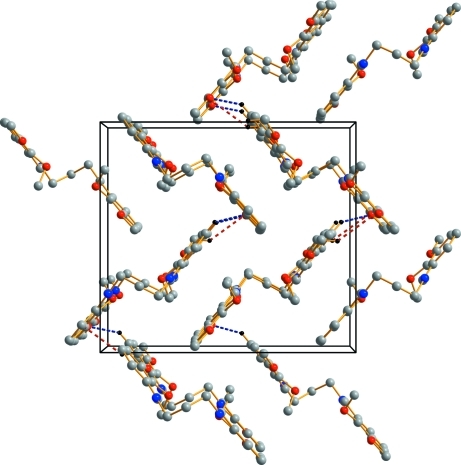
The packing of (I) viewed along b, with hydrogen bonds shown as blue dashed lines and C—H···π interaction as red dashed lines.

## supplementary crystallographic information

### Comment

Schiff bases range among the most chelating ligands found in the field of coordination chemistry (Morshedi *et al.*, 2009*a*,b). Their compounds with transition metals have wide applications in the field of magnetism (Gao *et al.*, 2003) and catalysis (Ardizzoia *et al.*, 2009). The title compound  has been studied as an extension of our work on the structural characterization of bidentate Schiff base compounds with di- and trimethoxy benzaldehyde (Khalaji *et al.*, 2007 and Dehno Khalaji *et al.*, 2009).

The molecule of the title compound is shown in Fig.1. All bond lengths and angles are comparable with those observed in similar compounds (Khalaji *et al.*, 2007 and Dehno Khalaji *et al.*, 2009). The C7=N1 and C11=N2 bond lengths of 1.264 (2) and 1.263 (2) Å, respectively, conform to the value for a double bonds while C8—N1 and C10—N2 bond lengths of 1.458 (2) and 1.458 (2) Å, respectively, conform to the value for single bonds. The molecule displays an E configuration around the C=N double bond. The azomethine groups are coplanar with the aromatic rings. The dihedral angle between two phenyl rings is 8.16 (8)°. The neigbouring molecules are interconnected by C—H···O hydrogen bonds to infinite chains. These chains are linked by C—H···π interactions into sheets (Fig. 2).

### Experimental

2,3-Dimethoxybenzaldehyde (0.2 mmol) and 1,3-propanediamine (0.1 mmol) were dissolved in ethanol (50 ml). The mixture was stirred at room temperature for 1 h to give a clear solution. Suitable crystals of the title compound for X-ray study were formed by slow evaporation of the solvent over 8 days at room temperature (Yield 85%).

### Refinement

All hydrogen atoms were discernible in difference Fourier maps and could be refined to reasonable geometry. According to common practice they were nevertheless kept in ideal positions during the refinement. The isotropic atomic displacement parameters of hydrogen atoms were set to 1.2**U*_eq_ of the parent atom.

### Crystal data

**Table:**

C_21_H_26_N_2_O_4_	*F*(000) = 792
*M**_r_* = 370.4	*D*_x_ = 1.253 Mg m^−^^3^
Orthorhombic, *P**n**a*2_1_	Cu *K*α radiation, λ = 1.54184 Å
Hall symbol: P 2c -2n	Cell parameters from 24214 reflections
*a* = 15.3079 (3) Å	θ = 3.2–62.3°
*b* = 9.2915 (2) Å	µ = 0.71 mm^−^^1^
*c* = 13.8059 (3) Å	*T* = 120 K
*V* = 1963.66 (7) Å^3^	Irregular shape, colorless
*Z* = 4	0.51 × 0.31 × 0.20 mm

### Data collection

**Table:**

Oxford Diffraction Xcalibur diffractometer with an Atlas (Gemini ultra Cu) detector	1623 independent reflections
Radiation source: X-ray tube	1616 reflections with *I* > 3σ(*I*)
mirror	*R*_int_ = 0.021
Detector resolution: 20.7567 pixels mm^-1^	θ_max_ = 62.4°, θ_min_ = 5.6°
Rotation method data acquisition using ω scans	*h* = −17→17
Absorption correction: multi-scan (*CrysAlis PRO*; Oxford Diffraction, 2009)	*k* = −10→10
*T*_min_ = 0.628, *T*_max_ = 0.871	*l* = −15→15
26538 measured reflections	

### Refinement

**Table:**

Refinement on *F*^2^	H-atom parameters constrained
*R*[*F*^2^ > 2σ(*F*^2^)] = 0.025	Weighting scheme based on measured s.u.'s *w* = 1/[σ^2^(*I*) + 0.0016*I*^2^]
*wR*(*F*^2^) = 0.077	(Δ/σ)_max_ = 0.038
*S* = 1.97	Δρ_max_ = 0.10 e Å^−^^3^
1623 reflections	Δρ_min_ = −0.09 e Å^−^^3^
244 parameters	Extinction correction: B–C type 1 Lorentzian isotropic (Becker & Coppens, 1974)
0 restraints	Extinction coefficient: 2800 (1000)
105 constraints	

### Special details

**Table:**

Experimental. CrysAlisPro (Oxford Diffraction, 2009) Version 1.171.33.34d (release 27-02-2009 CrysAlis171 .NET) Empirical absorption correction using spherical harmonics, implemented in SCALE3 ABSPACK scaling algorithm.
Refinement. The refinement was carried out against all reflections. The conventional *R*-factor is always based on *F*. The goodness of fit as well as the weighted *R*-factor are based on *F* and *F*^2^ for refinement carried out on *F* and *F*^2^, respectively. The threshold expression is used only for calculating *R*-factors *etc*. and it is not relevant to the choice of reflections for refinement.The program used for refinement, Jana2006, uses the weighting scheme based on the experimental expectations, see _refine_ls_weighting_details, that does not force *S* to be one. Therefore the values of *S* are usually larger than the ones from the *SHELX* program.

### Fractional atomic coordinates and isotropic or equivalent isotropic displacement parameters (Å^2^)

**Table:**

	*x*	*y*	*z*	*U*_iso_*/*U*_eq_	
O1	0.26797 (8)	0.00499 (13)	0.18586 (12)	0.0306 (4)	
O2	0.18047 (7)	−0.18392 (11)	0.07546 (13)	0.0282 (3)	
O3	0.46602 (7)	0.01788 (12)	0.27381 (11)	0.0315 (4)	
O4	0.57090 (7)	−0.12117 (13)	0.40069 (13)	0.0348 (4)	
N1	0.22853 (8)	0.43567 (14)	0.17846 (14)	0.0279 (4)	
N2	0.46937 (8)	0.45543 (14)	0.24531 (14)	0.0281 (4)	
C1	0.18698 (9)	0.20195 (17)	0.11887 (15)	0.0239 (4)	
C2	0.20316 (10)	0.05443 (16)	0.12523 (15)	0.0239 (4)	
C3	0.16066 (9)	−0.04171 (17)	0.06289 (16)	0.0246 (4)	
C4	0.10498 (10)	0.01093 (17)	−0.00881 (15)	0.0274 (5)	
C5	0.09133 (10)	0.15967 (17)	−0.01601 (15)	0.0292 (5)	
C6	0.13056 (11)	0.25368 (17)	0.04693 (16)	0.0272 (5)	
C7	0.23004 (10)	0.30040 (17)	0.18825 (14)	0.0246 (4)	
C8	0.27152 (10)	0.52016 (17)	0.25365 (14)	0.0263 (5)	
C9	0.34446 (10)	0.61189 (16)	0.21099 (14)	0.0262 (5)	
C10	0.41895 (11)	0.52360 (17)	0.16833 (15)	0.0277 (5)	
C11	0.47382 (10)	0.31972 (17)	0.24671 (15)	0.0253 (5)	
C12	0.52555 (9)	0.24424 (17)	0.32177 (14)	0.0244 (4)	
C13	0.52359 (10)	0.09464 (17)	0.32989 (15)	0.0249 (4)	
C14	0.57636 (10)	0.02498 (19)	0.39893 (16)	0.0275 (5)	
C15	0.62910 (10)	0.10661 (19)	0.46004 (15)	0.0301 (5)	
C16	0.62956 (11)	0.25595 (19)	0.45242 (15)	0.0303 (5)	
C17	0.57829 (10)	0.32443 (18)	0.38485 (15)	0.0272 (5)	
C18	0.23861 (13)	−0.0640 (2)	0.27226 (15)	0.0402 (6)	
C19	0.14018 (12)	−0.28468 (19)	0.01141 (16)	0.0327 (5)	
C20	0.50416 (12)	−0.0730 (2)	0.20153 (18)	0.0396 (6)	
C21	0.62707 (12)	−0.1948 (2)	0.46665 (17)	0.0400 (6)	
H4	0.076366	−0.053919	−0.052675	0.0329*	
H5	0.053814	0.196398	−0.065946	0.035*	
H6	0.119366	0.355051	0.041722	0.0327*	
H7	0.260269	0.26004	0.242784	0.0295*	
H8a	0.295303	0.456715	0.301782	0.0315*	
H8b	0.229431	0.581308	0.284681	0.0315*	
H9a	0.32086	0.674064	0.162003	0.0314*	
H9b	0.366972	0.675138	0.259983	0.0314*	
H10a	0.395548	0.450955	0.126235	0.0333*	
H10b	0.456428	0.58522	0.131065	0.0333*	
H11	0.443276	0.264808	0.198515	0.0303*	
H15	0.66515	0.059858	0.507446	0.0361*	
H16	0.666035	0.31172	0.494737	0.0364*	
H17	0.5786	0.427575	0.380869	0.0327*	
H18a	0.288071	−0.09841	0.308189	0.0482*	
H18b	0.206536	0.003592	0.311013	0.0482*	
H18c	0.201483	−0.14353	0.255709	0.0482*	
H19a	0.156576	−0.380645	0.029678	0.0393*	
H19b	0.077848	−0.274903	0.015211	0.0393*	
H19c	0.159047	−0.266242	−0.053739	0.0393*	
H20a	0.4592	−0.108672	0.159426	0.0475*	
H20b	0.545747	−0.018776	0.164399	0.0475*	
H20c	0.533122	−0.15241	0.23229	0.0475*	
H21a	0.615974	−0.296407	0.463211	0.0479*	
H21b	0.686855	−0.176274	0.449786	0.0479*	
H21c	0.616154	−0.16141	0.531323	0.0479*	

### Atomic displacement parameters (Å^2^)

**Table:**

	*U*^11^	*U*^22^	*U*^33^	*U*^12^	*U*^13^	*U*^23^
O1	0.0289 (6)	0.0321 (7)	0.0309 (6)	0.0034 (4)	−0.0083 (5)	0.0007 (5)
O2	0.0280 (6)	0.0220 (6)	0.0346 (6)	−0.0006 (4)	−0.0038 (5)	−0.0025 (5)
O3	0.0245 (6)	0.0338 (6)	0.0362 (7)	−0.0033 (4)	−0.0073 (5)	−0.0013 (5)
O4	0.0298 (6)	0.0339 (6)	0.0405 (7)	−0.0035 (4)	−0.0091 (5)	0.0143 (6)
N1	0.0261 (7)	0.0265 (7)	0.0310 (8)	0.0016 (5)	−0.0001 (6)	−0.0017 (6)
N2	0.0267 (7)	0.0287 (7)	0.0288 (7)	0.0025 (5)	0.0028 (6)	−0.0020 (6)
C1	0.0185 (7)	0.0264 (8)	0.0268 (8)	0.0012 (6)	0.0048 (6)	−0.0005 (7)
C2	0.0185 (7)	0.0281 (8)	0.0250 (8)	0.0017 (6)	0.0011 (6)	−0.0002 (6)
C3	0.0194 (7)	0.0245 (8)	0.0298 (8)	−0.0010 (5)	0.0052 (7)	−0.0003 (7)
C4	0.0179 (7)	0.0347 (9)	0.0297 (9)	−0.0020 (6)	0.0000 (7)	−0.0032 (7)
C5	0.0225 (8)	0.0348 (8)	0.0303 (10)	0.0045 (6)	−0.0027 (7)	0.0020 (7)
C6	0.0237 (8)	0.0259 (8)	0.0322 (8)	0.0035 (6)	0.0025 (6)	0.0011 (7)
C7	0.0219 (7)	0.0269 (8)	0.0250 (8)	0.0005 (6)	0.0021 (6)	−0.0006 (6)
C8	0.0286 (9)	0.0236 (7)	0.0266 (8)	0.0038 (6)	0.0008 (7)	−0.0028 (7)
C9	0.0308 (8)	0.0219 (8)	0.0258 (8)	0.0023 (6)	0.0002 (6)	−0.0004 (6)
C10	0.0306 (9)	0.0268 (8)	0.0257 (8)	0.0014 (6)	0.0025 (7)	0.0002 (7)
C11	0.0214 (8)	0.0302 (8)	0.0243 (8)	0.0007 (5)	0.0019 (6)	−0.0039 (7)
C12	0.0184 (7)	0.0313 (8)	0.0236 (8)	0.0013 (5)	0.0046 (6)	−0.0018 (7)
C13	0.0181 (7)	0.0327 (8)	0.0241 (8)	−0.0017 (6)	0.0010 (6)	−0.0002 (7)
C14	0.0213 (7)	0.0355 (8)	0.0256 (8)	−0.0001 (6)	0.0033 (6)	0.0032 (7)
C15	0.0198 (7)	0.0461 (10)	0.0243 (8)	0.0018 (7)	−0.0018 (6)	0.0025 (7)
C16	0.0229 (8)	0.0417 (9)	0.0264 (9)	0.0008 (6)	0.0011 (6)	−0.0086 (8)
C17	0.0195 (8)	0.0334 (9)	0.0289 (9)	0.0016 (6)	0.0038 (6)	−0.0073 (7)
C18	0.0560 (12)	0.0369 (10)	0.0277 (9)	0.0151 (8)	0.0010 (8)	0.0018 (7)
C19	0.0338 (9)	0.0256 (8)	0.0388 (9)	−0.0044 (7)	−0.0058 (7)	−0.0029 (7)
C20	0.0442 (10)	0.0393 (9)	0.0352 (10)	−0.0108 (8)	−0.0042 (8)	−0.0069 (8)
C21	0.0356 (10)	0.0442 (10)	0.0401 (10)	0.0030 (7)	−0.0057 (8)	0.0177 (9)

### Geometric parameters (Å, °)

**Table:**

O1—C2	1.377 (2)	C9—H9a	0.96
O1—C18	1.427 (2)	C9—H9b	0.96
O2—C3	1.3668 (18)	C10—H10a	0.96
O2—C19	1.428 (2)	C10—H10b	0.96
O3—C13	1.373 (2)	C11—C12	1.481 (3)
O3—C20	1.432 (3)	C11—H11	0.96
O4—C14	1.361 (2)	C12—C13	1.395 (2)
O4—C21	1.427 (3)	C12—C17	1.402 (2)
N1—C7	1.264 (2)	C13—C14	1.407 (3)
N1—C8	1.458 (2)	C14—C15	1.392 (3)
N2—C10	1.458 (2)	C15—C16	1.392 (2)
N2—C11	1.263 (2)	C15—H15	0.96
C1—C2	1.396 (2)	C16—C17	1.375 (3)
C1—C6	1.401 (3)	C16—H16	0.96
C1—C7	1.479 (2)	C17—H17	0.96
C2—C3	1.401 (2)	C18—H18a	0.96
C3—C4	1.395 (3)	C18—H18b	0.96
C4—C5	1.401 (2)	C18—H18c	0.96
C4—H4	0.96	C19—H19a	0.96
C5—C6	1.371 (3)	C19—H19b	0.96
C5—H5	0.96	C19—H19c	0.96
C6—H6	0.96	C20—H20a	0.96
C7—H7	0.96	C20—H20b	0.96
C8—C9	1.523 (2)	C20—H20c	0.96
C8—H8a	0.96	C21—H21a	0.96
C8—H8b	0.96	C21—H21b	0.96
C9—C10	1.523 (2)	C21—H21c	0.96
			
C2—O1—C18	115.54 (13)	N2—C11—C12	120.82 (17)
C3—O2—C19	117.35 (15)	N2—C11—H11	119.5897
C13—O3—C20	115.95 (13)	C12—C11—H11	119.5889
C14—O4—C21	116.94 (15)	C11—C12—C13	121.11 (15)
C7—N1—C8	116.79 (16)	C11—C12—C17	119.41 (14)
C10—N2—C11	118.25 (16)	C13—C12—C17	119.48 (16)
C2—C1—C6	119.40 (16)	O3—C13—C12	119.09 (15)
C2—C1—C7	119.17 (16)	O3—C13—C14	120.77 (15)
C6—C1—C7	121.43 (14)	C12—C13—C14	120.03 (16)
O1—C2—C1	119.59 (15)	O4—C14—C13	115.84 (16)
O1—C2—C3	119.70 (13)	O4—C14—C15	124.64 (17)
C1—C2—C3	120.35 (16)	C13—C14—C15	119.52 (16)
O2—C3—C2	115.82 (16)	C14—C15—C16	120.02 (17)
O2—C3—C4	124.38 (16)	C14—C15—H15	119.9905
C2—C3—C4	119.75 (14)	C16—C15—H15	119.9904
C3—C4—C5	119.16 (16)	C15—C16—C17	120.65 (17)
C3—C4—H4	120.42	C15—C16—H16	119.6771
C5—C4—H4	120.4205	C17—C16—H16	119.6769
C4—C5—C6	121.22 (17)	C12—C17—C16	120.28 (15)
C4—C5—H5	119.3896	C12—C17—H17	119.8593
C6—C5—H5	119.3903	C16—C17—H17	119.8611
C1—C6—C5	120.05 (15)	O1—C18—H18a	109.4709
C1—C6—H6	119.9753	O1—C18—H18b	109.4711
C5—C6—H6	119.9765	O1—C18—H18c	109.4709
N1—C7—C1	122.50 (17)	H18a—C18—H18b	109.4714
N1—C7—H7	118.7511	H18a—C18—H18c	109.4717
C1—C7—H7	118.7506	H18b—C18—H18c	109.4712
N1—C8—C9	110.91 (16)	O2—C19—H19a	109.4712
N1—C8—H8a	109.4708	O2—C19—H19b	109.4713
N1—C8—H8b	109.4707	O2—C19—H19c	109.4707
C9—C8—H8a	109.4715	H19a—C19—H19b	109.4714
C9—C8—H8b	109.4712	H19a—C19—H19c	109.4716
H8a—C8—H8b	107.9975	H19b—C19—H19c	109.4711
C8—C9—C10	113.38 (13)	O3—C20—H20a	109.4712
C8—C9—H9a	109.4711	O3—C20—H20b	109.4716
C8—C9—H9b	109.471	O3—C20—H20c	109.4718
C10—C9—H9a	109.4708	H20a—C20—H20b	109.471
C10—C9—H9b	109.4724	H20a—C20—H20c	109.4707
H9a—C9—H9b	105.2553	H20b—C20—H20c	109.4711
N2—C10—C9	110.39 (16)	O4—C21—H21a	109.4718
N2—C10—H10a	109.4708	O4—C21—H21b	109.4707
N2—C10—H10b	109.4703	O4—C21—H21c	109.4713
C9—C10—H10a	109.472	H21a—C21—H21b	109.4709
C9—C10—H10b	109.4717	H21a—C21—H21c	109.4715
H10a—C10—H10b	108.5415	H21b—C21—H21c	109.4711

### Hydrogen-bond geometry (Å, °)

**Table:**

*D*—H···*A*	*D*—H	H···*A*	*D*···*A*	*D*—H···*A*
C5—H5···O4^i^	0.96	2.59	3.411 (2)	143

Symmetry codes: (i) −*x*+1/2, *y*+1/2, *z*−1/2.
